# Effect of Short-Term Contact with C1–C4 Monohydric Alcohols on the Water Permeance of MPD-TMC Thin-Film Composite Reverse Osmosis Membranes

**DOI:** 10.3390/membranes9080092

**Published:** 2019-07-26

**Authors:** Jaime A. Idarraga-Mora, Michael A. Lemelin, Steven T. Weinman, Scott M. Husson

**Affiliations:** 1 Department of Chemical and Biomolecular Engineering, Clemson University, 127 Earle Hall, Clemson, SC 29634, USA; 2 Department of Chemical and Biological Engineering, The University of Alabama, Box 870203, Tuscaloosa, AL 35487, USA

**Keywords:** thin-film composite, alcohol contact, water permeance, active layer

## Abstract

In this paper, we discuss the effect of alcohol contact on the transport properties of thin-film composite reverse osmosis membranes. Five commercial membranes were studied to quantify the changes in water permeance and sodium chloride rejection from contact with five C1–C4 monohydric, alcohols. Water permeance generally increased without decreasing rejection after short-term contact. The extent of these changes depends on the membrane and alcohol used. Young′s modulus measurements showed decreased stiffness of the active layer after contacting the membranes with alcohol, suggesting plasticization. Data analysis using a dual-mode sorption model identified positive correlations of the initial water permeance, as well as the change in free energy of mixing between water and the alcohols, with the increase in water permeance after alcohol contact. We suggest that the mixing of water with the alcohols facilitates alcohol penetration into the active layer, likely by disrupting inter-chain hydrogen bonds, thus increasing the free volume for water permeation. Our studies provide a modeling framework to estimate the changes in transport properties after short-term contact with C1–C4 alcohols.

## 1. Introduction

Thin-film composite (TFC) reverse osmosis (RO) membranes comprise a non-woven fabric backing, a porous support layer, and an active layer that typically is produced via interfacial polymerization of a diamine (e.g., m-phenylenediamine, MPD) and a triacyl chloride (e.g., trimesoyl chloride, TMC) [1]. TFC membranes are the current standard for membrane-based pressure-driven seawater desalination. Furthermore, TFC membranes have been used to develop new osmotically-driven membrane processes such as forward osmosis (FO), pressure-retarded osmosis (PRO), osmotically-assisted reverse osmosis (OARO) [2], and pressure-assisted forward osmosis (PAFO). One difference between pressure-driven processes (such as RO) and osmotically-driven processes (such as OARO, PAFO, FO, and PRO) is that the latter suffer from detrimental internal concentration polarization in the support layer. One significant reason for low membrane performance in FO is incomplete support layer wetting [3].

Due to the hydrophobic nature of the porous support layer [4], some suggest soaking membranes in a short-chain alcohol to ensure wetting prior to use [5,6,7]. Alcohols used for wetting are often ethanol and 2-propanol (or isopropyl alcohol, IPA), and contact times range from minutes to days [5,7,8,9,10,11,12,13]. However, the water permeance and salt rejection properties of TFC membranes can change after contact with these alcohols [5,7,8,9,10,11,12,13].

Table S1 (Supplementary Materials) presents a literature review of previous investigations on alcohol treatment and its effects on membrane productivity and selectivity. From these studies, it is known that short chain (C1–C4) alcohols improve the transport properties of MPD-TMC-based TFC RO membranes [9]. It has been observed that different alcohols lead to different changes in water flux. However, the causes of this behavior have not been studied sufficiently [7,14,15]. In addition, it has been concluded that changes in the polymer active layer of these membranes are dependent on size of the alcohol, composition of the polymer, free volume in the polymer (especially before contact), and interactions between polymer and alcohol [12,16]. However, the characteristics of the active layer polymers that most enable changes in transport properties have not been determined [7,10,11,13]. Some authors suggest that dissolution of polymer chains from the active layer or other coatings results in increased membrane productivity [10,17]. It also has been proposed that swelling of the active layer occurs, which may disrupt inter-chain interactions, such as hydrogen bonding, creating space for water to penetrate into the membrane [14]. Positron annihilation spectroscopy (PAS) studies report contradictory findings on the changes in permeance and salt rejection that occur when contacting polyamide networks with C1–C4 alcohols [11,13]. Finally, the above-mentioned studies propose that polyamide-alcohol interactions contribute to the improvement in transport properties. However, Aharoni [18] showed that interactions between fully-aromatic polyamides and methanol are low, and are estimated to increase with alcohol chain length.

This paper contributes to the understanding of the effects of short-chain (C1–C4) monohydric alcohols on the transport properties of commercial TFC RO membranes with an MPD-TMC-based active layer and different initial transport properties. Our hypothesis is that multiple previously observed effects occur when contacting active layers with alcohols, related to the miscibility of the alcohol in water, the polyamide interactions with alcohol, and the initial condition of the polymer before contact with alcohol. Differences in active layer chemistry, coatings, morphology, surface roughness, alcohol affinity, and stiffness were studied before and after treatment with alcohols and water. Changes in active layer transport properties were measured for five short-chain alcohols and five commercial TFC RO membranes. A simple dual-mode sorption mathematical model was used to differentiate contributions of each active layer and each alcohol to the changes in transport properties. Our studies provide a modeling framework to estimate the changes in transport properties after short-term contact with short-chain alcohols that is especially useful when selecting conditions for wetting the support layer of TFC membranes for osmotically-driven membrane processes.

## 2. Theory

The internal volume (free volume) of the polyamide layer of TFC membranes has been investigated by Kim et al. using PAS. PAS revealed that polyamide layers of MPD-TMC-based TFC membranes have two main pore types: aggregate (0.35–0.45 nm) and network pores (0.21–0.24 nm) [19]. They also found that adding small amounts of dimethyl sulfoxide to the aqueous phase during synthesis increased the content of aggregate pores compared to network pores, which resulted in higher water passage through the membrane without considerable loss of salt rejection [19,20]. Similarly, Aharoni observed that contacting rigid polyamide networks with a swelling agent (*N*,*N*-dimethylacetamide, DMAc) until equilibrium was established, followed by immersion of the polymer into non-solvent mixtures (DMAc-methanol, DMAc-acetone), resulted in a polyamide sample with larger bulk volume compared to the same specimen initially [18,21]. On the other hand, Guo and Barbari used a dual-mode sorption model to describe the swelling of a glassy polymer [22,23]. They found that internal space was formed upon sorption of a penetrant and these holes would remain after desorption [22]. We adapted this dual-mode sorption model to help explain the changes seen in water permeance for the different fully-aromatic polyamide active layers (MPD-TMC chemistry) using short-chain alcohols at constant treatment time. 

From the dual-mode sorption model (Equation (1)), the concentration of water within the polymer ($c_{w,m,f}$) after alcohol penetration is the combination of the concentration of water dissolved in the polymer (*c_w,D_*) and the concentration of water in the newly generated pores (*c_w,H_*):

(1) $$c_{w,m,f}=c_{w,D}+c_{w,H}$$

If the water content in the polyamide layer is very low, then we can assume that the concentration of water in the internal volume is proportional to the activity (*a*) of the penetrant and the proportionality constant is the Henry’s constant of water ($K_{w}$). The number of generated pores by penetrant contact is assumed to be limited, and it can be represented as a Langmuir-isotherm type curve [24]. Equation (2) shows the water concentration in the polymer after alcohol contact can be redefined in terms of the activity of the penetrant (*a =* 1 for a pure liquid penetrant), the pre-existing capacity of the polymer (*C′_H_*) to create new pores, and the affinity of the penetrant with the polymer (*b*):

(2) $$c_{w,m,f}=K_{w}a+C_{H}^{\prime }\frac{ba}{1+ba}$$

Equation (3) shows that *K_w_* can be determined from the initial water permeance (*A*_0_) of the TFC membrane before pores are generated by the penetrant as:

(3) $$A_{0}=\frac{D_{w,m}\nu^{2}c_{w,m,0}}{\delta R_{u}T}K_{w}=A^{\prime }K_{w}$$

$c_{w,m,0}$ is the concentration of water in the solution in contact with the membrane, $D_{w,m}$ is the water diffusivity within the polymer, ν is the molar volume of water, δ is the membrane thickness, $R_{u}$ is the universal gas constant, and T is the temperature. The water diffusivity within the membrane is assumed to be dependent mostly on temperature (22 °C in this study). Therefore, it is treated as a constant during permeation tests before and after alcohol contact. Freger showed that changes in thickness due to ethanol swelling of TFC RO membranes are on the order of 5% [25]. Therefore, we assumed a constant thickness of 145 nm by averaging the reported values for SW30HR and XLE measured via ellipsometry and reported by Coronell and coworkers [26].

Finally, we can relate the initial water permeance, *A*_0_, with the resulting water permeance after contact with ethanol (*A_f_*) by substituting Equation (3) into Equation (2) to give Equations (9)–(14):

(4) $$c_{w,m,f}=\frac{A_{0}}{A\prime }a+C_{H}^{\prime }\frac{ba}{1+ba}$$

(5) $$A\prime c_{w,m,f}=A_{0}+A^{\prime }C_{H}^{\prime }\frac{b}{1+b}$$

(6) $$A_{f}=A_{0}+A^{\prime }C_{H}^{\prime }\frac{b}{1+b}$$

(7) $$A_{f}=A_{0}+K_{1}K_{2}$$

(8) $$K_{1}=A^{\prime }C_{H}^{\prime }$$

(9) $$K_{2}=\frac{b}{1+b}$$

$K_{1}$ is related to pre-existing properties of the active layer, whereas $K_{2}$ relates to the interactions between the alcohol and the active layer. The values for *K*_1_ and *K*_2_ were obtained by fitting the experimental data to Equation (7). Initially, this model suggests that to realize changes in the water permeance, it is necessary to have pre-existing capacity to generate holes and an affinity between the solvent and the active layer. The value of $K_{1}$ represents the maximum water permeance change that can be obtained after contact with an alcohol of high affinity for the established time. On the other hand, $K_{2}$ indicates the extent of the change in water permeance due to the interaction between the alcohol and the active layer, and it should be a value between 0 and 1.

## 3. Experimental

### 3.1. Materials

TFC membranes were provided by Dow Water & Process Solutions (Edina, MN, USA). Seawater desalination membranes SW30HRLE, SW30XLE, and SEAMAXX; and brackish water desalination membranes BW30XFR and XLE were used in this work. The following chemicals were used as received from ThermoFisher Scientific (Waltham, MA, USA): methanol (Optima® for HPLC, MeOH), ethanol (anhydrous, 200 proof, >99.5%, Acros Organics, EtOH), and 2-propanol (isopropanol, IPA, Certified ACS Plus). The following chemicals were used as received from Sigma-Aldrich (St. Louis, MO, USA): 1-propanol (ACS reagent, >99.5%, 1-PrOH), 1-butanol (for molecular biology, >99%, 1-BtOH), and sodium chloride (anhydrous, ACS reagent, >99%). The following chemicals were used as received from VWR International (Radnor, PA, USA): potassium chloride (BDH, ACS grade) and sodium hydroxide (Amresco, ACS grade). Deionized (DI) water (resistivity = 18 MΩ⋅cm) was obtained using a Milli-Q water purification system (EMD Millipore, Billerica, MA, USA).

### 3.2. TFC Membrane Characterization

Attenuated total reflectance Fourier-transform infrared spectroscopy (ATR-FTIR) was used to confirm the chemistry of the active layer of the membranes studied and to identify membranes with coatings. The instrument used was a Thermo Scientific bench-scale Nicolet iS50R FT-IR (ThermoFisher Scientific, Waltham, MA, USA) equipped with a Specac Golden Gate Diamond ATR (Specac Incorporated, Fort Washington, PA, USA). Scan settings were 128 scans with 4 cm^−1^ resolution and beam auto-gain. The as-received membranes were rinsed with DI water to remove any protective coating and humectant and vacuum dried for more than 24 h before testing.

Streaming potential (zeta potential) measurements were used to identify the presence of coatings by determining the surface charge of the membrane active layer. The instrument used was an Anton Paar SurPass equipped with a clamping cell. The control and data logging were done using Anton Paar Visiolab software (Anton Paar GmbH, Graz, Austria). A streaming channel was created by placing two membrane coupons with the active layers facing each other, and separated by two spacers. Both coupons were from the same membrane reference that had been rinsed with DI water. The streaming channel length was 25 mm. Measurements started at pH 5.6 ± 0.1 by using a 1 mM solution of potassium chloride. The pH was increased by adding 0.1 mL aliquots of 0.1 M sodium hydroxide solution to the solution reservoir until the pH was above 9. The software reported the measured pH and calculated zeta potential using the Fairbrother–Mastin approach. Reported uncertainties represent the standard deviation from four measurements using the same streaming channel.

X-ray photoelectron spectroscopy (XPS) was used to measure the atomic composition of the membrane surface to identify coatings. Membrane samples were washed thoroughly with DI water, soaked in DI water for 24 h, and then vacuum dried for 8 h before testing. The instrument used was a VersaProbe III Scanning XPS Microprobe (Physical Electronics Inc., Chanhassen, MN, USA) with a monochromatic Al Kα source (1,486.7 eV). A survey scan was done from 0 to 1,100 eV using a pass energy of 224 eV and step of 0.8 eV. For quantitative analysis of carbon, nitrogen, and oxygen, higher resolution scans were performed. Scans were done from 278 to 298 eV for C1s, from 391 to 411 eV for N1s, and from 523 to 543 eV for O1s, all using a pass energy of 69 eV and 0.125 eV steps. To minimize charging, we used an electron flood gun and low voltage Ar ion gun at 3 eV. The reported results are the average of two survey scans. For all analyses, we used a 100 µm diameter, 25 W beam to scan an area of 500 µm × 500 µm. Three different areas were analyzed. 

Scanning electron microscopy (SEM) was used to image the top surface of the membranes. Images were taken for the following membrane types: as received and rinsed with DI water, after contact with ethanol for 5 min, after contact with ethanol and then water (5 min each), and after being tested in RO mode. Samples were sputtered-coated at 60 mbar with gold-palladium using an Anatech Hummer 6.5 (Anatech Limited, Denver, NC, USA) coater. A Hitachi S4800 field emission microscope (Hitachi Limited, Tokyo, Japan) was used to capture images using an accelerating voltage of 10 kV.

Captive air bubble contact angle (CB-CA) measurements were performed on each membrane active layer using different alcohols and water to determine their chemical affinity. Membranes were rinsed with DI water to remove protective coatings and humectants and were carefully pat dried with lint-free Kimwipes. The instrument used to capture instantaneous images was a Krüss DSA 10 Mk2 goniometer (Krüss GmbH, Hamburg, Germany) with Drop Shape Analysis software (ver. 1.80.0.2, Krüss GmbH, Hamburg, Germany). The images were analyzed using the Low Bond Axisymmetric Drop Shape Analysis (LBADSA) plug-in (ver. March 2005) [27] for ImageJ (National Institutes of Health, Bethesda, MD, USA). It was assumed that the captive bubbles followed an axisymmetric profile that follows the Young–Laplace equation. Therefore, capillary constants for the alcohols were calculated using Equation (10) (surface tension reported in Table S2) [27]. Six measurements were done per membrane per alcohol.

(10) $$\mathrm{capillary}\;\mathrm{constant}=\frac{\mathrm{density}\times \mathrm{gravity}}{\mathrm{surface}\;\mathrm{tension}}$$

Atomic force microscopy (AFM) was used to measure the Young′s Modulus of the active layer before and after contact with each alcohol using force-volume contact mode. Control samples were rinsed with DI water to remove any protective coating and humectant, immersed in DI water for 5 min, pat dried, mounted on a glass slide, and tested immediately. Test samples were rinsed with DI water, immersed in the desired alcohol for 5 min, re-immersed in DI water for 5 min, and mounted on a petri dish. The petri dish was filled with enough water to cover the membrane surface (2–4 mL), and the membrane was tested immediately. The instrument used was a Bioscope AFM (Bruker Inc., Billerica, MA, USA) with a Nanoscope IIIa controller and Nanoscope version 5.32R1 software (Bruker Inc., Billerica, MA, USA). Standard AFM probes HQ:NSC16/Al BS from MicroMasch (Watsonville, CA, USA) were used for all measurements. For force-volume measurements, 256 measurements were taken over a scan area of 10 μm × 10 μm. At least 3 different spots were analyzed per control sample. To calculate the Young′s Modulus, the force-volume curves were analyzed using the Sneddon (Conical) model including the adhesion force. The active layer Poisson′s ratio was assumed to be 0.39 [28], and the minimum and maximum force fit boundaries were set to 5% and 100%.

AFM was used to evaluate the roughness of the surface of each membrane tested, before and after contact with different alcohols, using imaging tapping mode. For imaging, 512 samples were taken per line over an area of 5 μm × 5 μm at a scan rate of 1 Hz. At least 3 different spots were analyzed per membrane per alcohol.

### 3.3. TFC Membrane Performance Testing

Water permeance (*A*) and salt rejection (*R*) were measured before and after membrane contact with the short-chain alcohols. A membrane coupon was cut from a membrane roll and soaked in DI water for 5 min to remove any protective coating and humectant. The entire permeation cell assembly (Sterlitech HP4750, Sterlitech Corporation, Kent, WA, USA) was rinsed with DI water before assembly. The test cell stirrer was placed in the test cell and the cell was filled with a 2000 ppm sodium chloride feed solution (osmotic pressure of 1.7 bar). The cell was set on a stir plate and the stirring speed was set to 120 RPM. 

The system was pressurized to 17.2 bar using air. The control valve was opened slowly to increase the pressure by approximately 0.07–0.14 bar per second. The system pressure remained constant for the duration of the permeation test. Measurements were taken 30 min after the permeation rate became constant. The mass of permeate (*m_P_*) was recorded for a known time and the water flux was calculated using Equation (11), where *ρ_w_* is the density of the permeate, and *A_c_* is the membrane active area, 14.6 cm^2^.

(11) $$J_{w}=\frac{m_{P}}{t\rho_{w}A_{c}}$$

To evaluate the change in permeance due to alcohol contact, the test cell was depressurized and disassembled from the air supply, the remaining sodium chloride solution was removed, and the cell was rinsed thoroughly with DI water. Then, 50 mL of alcohol was poured into the cell and left for 5 min or 2 h. When the allotted time had passed, the alcohol was removed from the cell, the cell was rinsed thoroughly with DI water, and the permeation procedure described above was repeated using the same membrane coupon. Finally, salt rejection was evaluated by measuring the conductivity of the feed and permeate samples using a Sensorex Corporation (Garden Grove, CA, USA) CX100 conductivity meter and CS150TC probe and applying a linear calibration between conductivity and sodium chloride concentration to determine feed and permeate salt concentrations (*c_F_* and *c_P_*). The water permeance and salt rejection were calculated using Equations (12) and (13). The osmotic pressure difference (Δ*π*) was estimated using the Van′t Hoff Equation (Equation (14)) with a factor *i* = 2 for sodium chloride. This procedure was repeated at least three times for each membrane type. The same procedure was done using 50 mL of DI water before the second permeation test as a control experiment.

(12) $$A=\frac{J_{w}}{\left(\Delta P-\Delta \pi \right)}$$

(13) $$R=1-\frac{c_{P}}{c_{F}}$$

(14) $$\Delta \pi =iRT\left(c_{F}-c_{P}\right)$$

## 4. Results and Discussion

**Figure 1:**
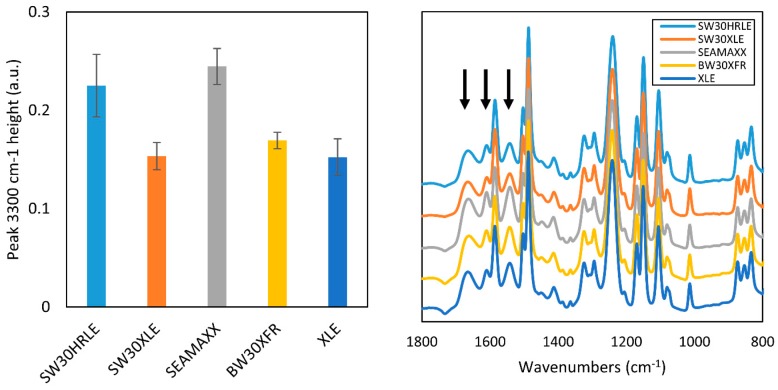
ATR-FTIR spectra of the TFC membranes studies. (**Left**) The peak height of characteristic –OH peak of membrane coatings at 3300 cm^−1^. (**Right**) Characteristic peaks of MPD-TMC-based polyamide layers are highlighted (**black arrows**).

Initially, all membranes were characterized using ATR-FTIR, and Figure 1 shows the IR spectra. All the membranes showed peaks at 1660 cm^−1^ (amide I band), 1610 cm^−1^ (aromatic amide), and 1540 cm^−1^ (amide II band), assignable to a fully aromatic polyamide chemical structure [29]. In addition, the absence of dominant peaks at 1630 and 1730 cm^−1^ indicates that the polyamide is not semi-aromatic (like some piperazine-based nanofiltration TFC membranes) [29]. Therefore, we concluded that all the polyamide layers of the membranes used in this study were made by the reaction of m-phenylenediamine (MPD) and trimesoyl chloride (TMC), which would yield a fully aromatic polyamide structure. On the other hand, Figure 1 data also reveal that the SW30HRLE and SEAMAXX membranes have increased peak heights at 3300 cm^−1^ assignable to –OH groups. These observations suggest that the SW30HRLE and SEAMAXX membranes have a coating, by comparison to the non-coated XLE membrane [30].

**Figure 2:**
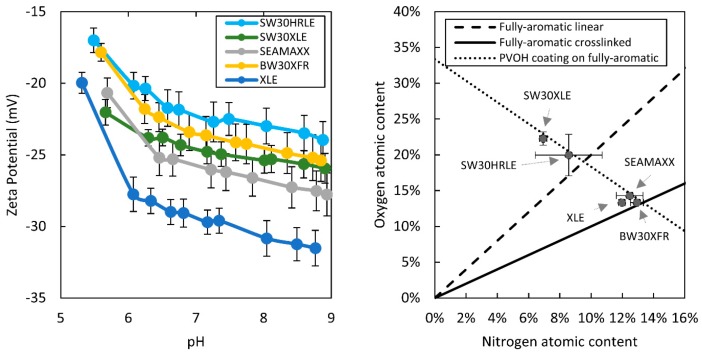
(**Left**) Zeta potential measurement data. (**Right**) Oxygen and nitrogen atomic content on the surface of the membranes measured via XPS.

To further assess whether the membranes were coated, we performed streaming potential and XPS measurements of the membranes. Streaming potential experiments were chosen because the presence of a neutral charge coating will reduce the zeta potential by reducing the net electrical charge contained within the region bounded by the slipping plane. XPS experiments provide information on surface composition. Figure 2 (left) shows the zeta potential of the membranes studied over a pH range from 5.5 to 9. The non-coated XLE membrane has the most negative charge at pH 7–9 (seawater) compared to the four other membranes. Furthermore, SW30HRLE shows the least negative surface charge over the same range. Figure 2 (right) shows the oxygen and nitrogen atomic composition of the membrane surfaces (carbon content is provided in Table S3). SW30HRLE and SW30XLE oxygen content is higher than what a theoretical linear MPD-TMC membrane would have, suggesting that an additional coating layer is present. The lines plotted in the figure represent theoretical layers comprising combinations of PVOH and MPD-TMC polyamide. SW30HRLE and SW30XLE have compositions that fall along the PVOH coating line, which supports that PVOH is the coating applied. The rest of the membranes have oxygen contents that fall within the expected range for an almost fully cross-linked MPD-TMC polyamide, further supporting that they have no coating or that their coatings have similar composition to the polyamide layer. These observations support the idea that SW30HRLE has a coating, most likely PVOH, whereas XLE is non-coated. A similar observation has been reported elsewhere [29,31]. We cannot rule out the possibility of a coating on the other membranes based on the reduced surface charge. However, it seems unlikely to be exclusively PVOH based on the concordance of FTIR and XPS data. These observations were used for the data analysis discussed below. 

### 4.1. Active Layer Characteristics in Contact with Short-Chain Alcohols and Water

Characterization techniques were used to investigate if there were changes in the polyamide layer after contact with different short-chain alcohols. Firstly, SEM was used to visualize the morphology changes of the polyamide layer. Figures S1 and S2 show SEM images at 2k and 10k magnification of the top surface of the TFC membranes after different treatments. Polyamide layers from seawater desalination membranes SW30HRLE and SW30XLE showed a ridge-and-valley structure, whereas the other membranes showed a flattened structure. Xu et al. fabricated fully aromatic polyamide layers and noticed that by changing monomer concentrations and ratios it is possible to obtain either a ridge-and-valley or a flatter polyamide layer structure [32]. Therefore, we believe that differences in the polyamide layer morphologies between membranes depend on the fabrication process and not the monomers used to fabricate them. No visible differences were seen when analyzing the polyamide layer images at different magnifications after alcohol treatments. Previous AFM studies have reported changes in the polyamide layer morphology by contacting it with ethanol [8,33]. Therefore, we believe that the vacuum-dry conditions of the SEM measurements do not allow for visualization of these changes, indicating that internal water content in hydrated layers can be the origin of the morphology changes seen by AFM.

AFM was used to quantify the change in the surface roughness of the polyamide layers after contact with different short-chain alcohols. Figure S3 shows the relative change in root-mean-(RMS) surface roughness of the polyamide top surface after immersion in the different alcohols compared to the surface roughness evaluated after immersion in water (Table S4). The results show that the average membrane surface roughness increases after contact with short-chain alcohols, except in the case of IPA, in which the average change was 0%. Liu and coworkers [8] also observed an increase in membrane surface roughness after contact with ethanol for 5 min. However, they also obtained an increase in roughness using a 50% IPA aqueous solution. The estimated solubility parameter of the aromatic polyamide solubility is 23 MPa^1/2^ [18], which is more similar to IPA (23.6 MPa^1/2^) than methanol (29.7 MPa^1/2^) or water (47.9 MPa^1/2^) [34]. This implies that polyamide-methanol interactions are less favorable than polyamide-IPA interactions. Nevertheless, larger roughness changes were observed with methanol, suggesting that changes in the polyamide layer structure after contact with alcohols cannot be explained exclusively on the basis of polyamide-alcohol interactions.

Figure S4 shows the Young′s modulus of the polyamide layer top surface dried and in water after contact with different alcohols for 5 min. The results show a statistically significant decrease (at least 90% confidence interval) in Young’s modulus for the SW30HRLE, SEAMAXX, and BW30XFR membranes after alcohol contact, and for the XLE membrane after ethanol, IPA, and 1-butanol contact. Previous work from our group suggests that plasticization occurs on TFC membranes when in contact with glycerol (a short chain polyhydric alcohol) solutions [35]. Shin et al. [16] also reported the reduction of surface modulus after contact of MPD-TMC polyamide layers with different solvents, such as ethanol and IPA. By comparing the dry samples with a sample tested in water after treatment, the reduction in Young′s modulus of the membrane surface (confidence interval above 90%) is more evident. This result is due to the increased water content within the polyamide layer after contact with water. 

Captive bubble contact angle (CB-CA) was used to further evaluate the interactions between the polyamide layer and short-chain alcohols. Figure S5 shows the contact angle results. The CB-CA measurements confirmed that the affinity between the polyamide layer of the membranes and short-chain alcohols is significantly higher (lower contact angle) than water. In general, methanol showed a higher contact angle compared to the other alcohols. However, it was not possible to clearly differentiate the effects of the rest of the alcohols tested, nor to differentiate between the membranes studied by their contact angle with a particular alcohol. Higher affinity towards the active layer would facilitate swelling of the active layer by alcohol, consistent with the plasticization observed when putting the membranes in contact with ethanol and IPA. 

### 4.2. Effect of Short-Chain Alcohol Contact on TFC Membrane Transport Properties

Permeation experiments were conducted to evaluate the effect of contact time and alcohol type on TFC membrane water permeance and salt rejection. We used a direct-flow filtration setup for these measurements. While this method is not ideal for estimating the salt rejection, it enabled the collection of data and analysis of multiple membranes and alcohols within the project timeline and budget. Because our goal in this study was to compare the effect of multiple alcohols on multiple active layers composed of substantially the same material, we used the same testing conditions for all membranes. The brackish water condition was selected based on standardized conditions for membrane testing [5]. We chose to double the suggested hydrostatic pressure to minimize the effect of concentration polarization, by having a considerable difference between the hydrostatic driving force (17.2 bar) and the osmotic pressure barrier caused by 2000 ppm NaCl (1.7 bar). Changes are reported relative to the measured values of water permeance and salt rejection during the first step of our experiments, before contact with any alcohol, as shown in Table 1. The water permeance values were within the ranges reported by the manufacturer for membranes SW30HRLE, SEAMAXX, and XLE. However, the values for SW30XLE and BW30XFR were lower [36]. We attribute this to the reduction of free volume in the membrane active layer due aging that is known to occur [37,38]. These polyamide layers are non-equilibrium materials in a glassy state. They age over time, so the properties will change. We attempted to avoid aging effects during our studies. However, we are unable to verify that all membranes studied are of constant age. The NaCl rejection values were lower than reported [36] due to the nature of the direct-flow testing, which leads to detrimental concentration polarization. Nevertheless, our interest was to evaluate the changes in transport properties of the membranes upon short-term contact, and the direct-flow test simplified the experimental protocol to make such comparisons. 

**Figure 3:**
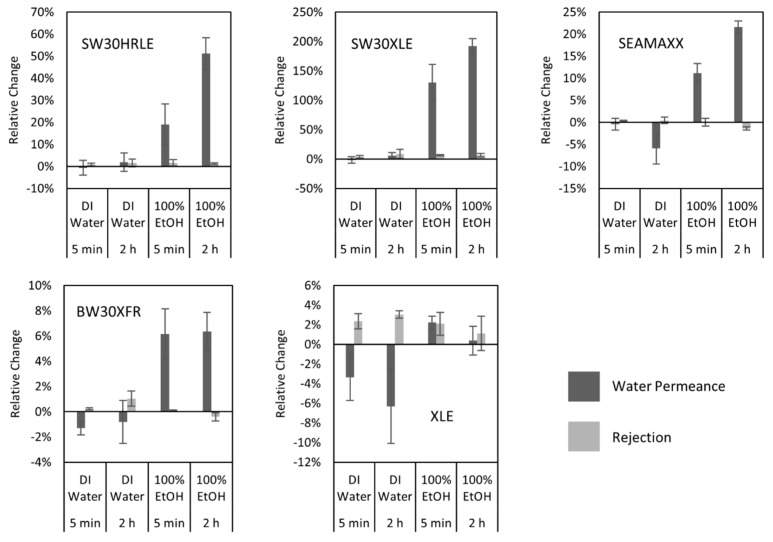
Relative change in water permeance (**dark**) and salt rejection (**light**) after water and ethanol contact for 5 min and 2 h for seawater (**top row**) and brackish water (**bottom row**) desalination membranes. Error bars represent one standard deviation for at least three measurements. Note that the y-axis scale is different for each membrane set.

Figure 3 shows the relative changes in water permeance and salt rejection of TFC membranes after contact with DI water and ethanol for 5 min and 2 h. Tables S5 and S6 report the results of the hypothesis test for each case with a confidence interval of 95%. Changes are not significant for seawater desalination membranes (SW30HRLE, SW30XLE, and SEAMAXX) after 5 min contact with DI water, whereas small decreases (<5%) were statistically significant for brackish water desalination membranes (BW30XFR and XLE). After 2 h of contact with DI water, XLE and SEAMAXX membranes showed a statistically significant decrease in permeance. No membrane showed a statistically significant decrease in salt rejection after 5 min or 2 h contact with DI water, but some showed a statistically significant increase of no greater than 5% in salt rejection. Reduced water flux after treatment with DI water can be explained by compaction of the membrane active and support layers [39,40], which is dependent of the initial free volume content of the active layer, and the compressive strength of the support layer [41]. The small decreases in water permeance in our control experiment were observed for the SEAMAXX, BW30XFR, and XLE membranes, which had the highest initial values; therefore, we believe that this is the result of the compaction of the high free volume of these active layers. 

Similarly, we evaluated the effect of ethanol contact on the membrane properties. Nearly all tested membranes showed a statistically significant increase in water permeance and no statistically significant decrease in salt rejection when contacted with ethanol. Exceptions were SEAMAXX, which showed a statistically significant decrease in salt rejection (1.5%) for 2 h contact with ethanol; and XLE, which showed no statistical change in both water permeance and salt rejection for 2 h contact with ethanol. In addition, seawater desalination membranes showed higher relative changes in permeance compared to brackish water desalination membranes after contact with ethanol, suggesting that the initial permeance influences the extent of the change observed after contact. The SW30XLE membrane, which had the lowest initial water permeance, showed the highest relative increment in this property. With these observations, we decided to expand our study to other short-chain alcohols establishing 5 min as the contact time for testing, because it appears to be sufficiently long to see water permeance changes without compromising the salt rejection of the membranes.

**Figure 4:**
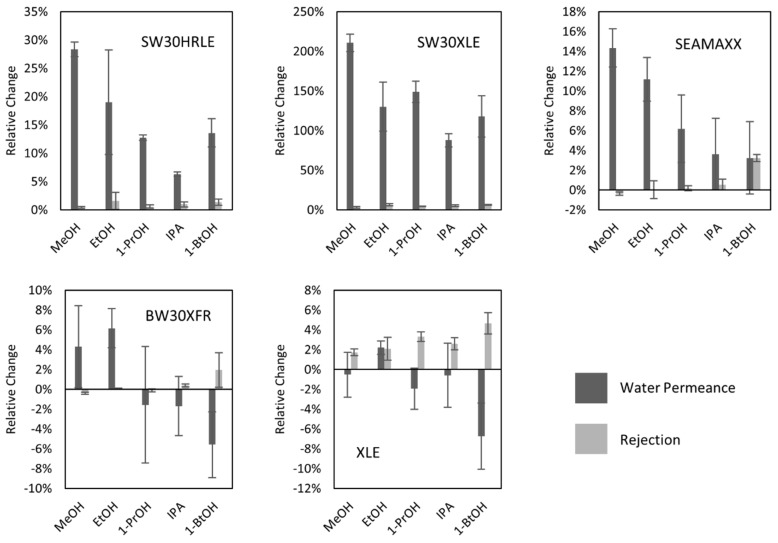
Relative change in water permeance (**dark**) and sodium chloride rejection (**light**) after contact with different alcohols for 5 min for seawater (**top row**) and brackish water (**bottom row**) desalination membranes. Error bars represent one standard deviation for at least three measurements.

Figure 4 shows the relative change in water permeance and salt rejection of TFC membranes after contact for 5 min with different short-chain alcohols. Table S7 reports the results of the hypothesis test for each case with a confidence interval of 95%. In nearly all cases, seawater desalination membranes showed increased water permeance after 5 min contact with short-chain alcohols (except SEAMAXX after contact with 1-butanol). The brackish water desalination membranes showed statistically significant increases in water permeance after contact with ethanol, statistically significant decreases in water permeance after contact with 1-butanol, and no statistical change in water permeance after contact with other alcohols. These results further suggest that the initial permeance plays a role in the permeance change and could be used as a predictor of the extent of the change after alcohol contact. On the other hand, the SEAMAXX and BW30XFR membranes showed a statistically significant decrease in salt rejection (<2%) when contacted with methanol. Methanol contact also yielded the highest statistically significant increases in water permeance for seawater desalination membranes. These observations indicate that short-term contact with methanol leads to a more open polyamide layer, which leads to higher water permeation and in some cases higher salt passage.

The increases in water permeance were higher for seawater desalination membranes with all the alcohols tested, compared to brackish water desalination membranes. This behavior is consistent with data in Figure 3 for ethanol contact. In addition, it was observed that methanol contact led to higher water permeance of seawater desalination membranes, whereas IPA and 1-butanol showed the lowest increases in water permeance for seawater desalination membranes. These findings suggest that the type of alcohol influences the extent of the change in transport properties of the membranes after alcohol contact.

**Figure 5:**
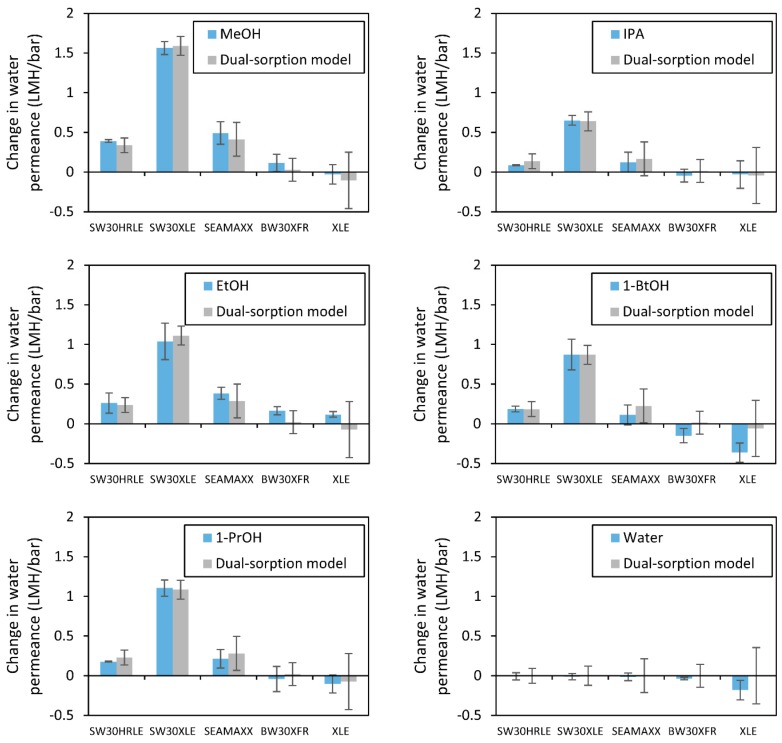
Change in water permeance (*A_f_–A*_0_) **(blue bars**; LMH/bar) after contact with different alcohols for 5 min. Gray bars indicate dual-mode sorption model fits of the data. Error bars represent one standard deviation of at least three measurements (**blue bars**) and error propagation of the initial water permeance (Table 1) in the model (**gray bars**).

Figure 5 shows the change in water permeance (*A_f_–A*_0_) after contact with each alcohol for each membrane tested. Gray bars represent dual-mode sorption model (Equation (7)) when fitted to the experimental data and the error bars include error propagation from the uncertainty (one standard deviation) in the initial water permeance. The experimental results fall within the uncertainty of the modeled results, suggesting that contacting short-chain alcohols with active layers of TFC membranes changes their internal volume that could be occupied by water. Table S8 shows the statistical analysis used to evaluate the model fit to the experimental data. The model and the experimental data show determination coefficients (*R*^2^) above 95% for the data grouped per alcohol, which indicates that the model correlates the differences between the different membranes. However, the model is less accurate predicting small changes in permeance such as the case of the brackish water desalination BW30XFR and XLE membranes. We attribute this discrepancy to the implicit assumption in the model that the active layer of each membrane is made of the same material, which is partially supported by the ATR-FTIR and XPS data, except in the case of the XLE membrane which is non-coated. 

**Figure 6:**
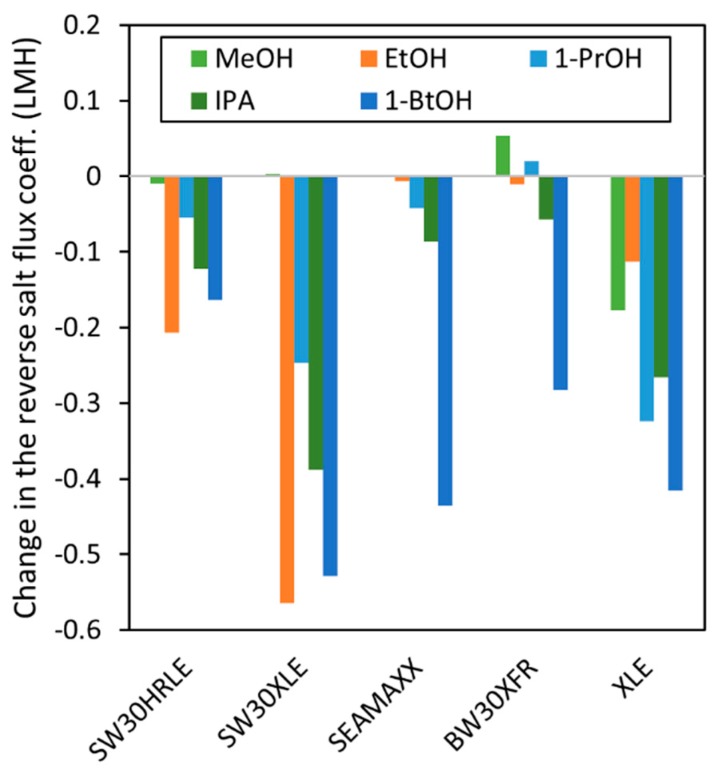
Change in the salt reverse flux coefficient *(B_f_–B*_0_*)* (calculated using Equation (15)) for each membrane after contact with different alcohols.

We further investigated the effect of alcohol contact on the studied membranes by calculating the salt reverse flux coefficient (*B*) using Equation (15) reported by Cath et al. [5]. The mass-transfer coefficient (*k*) estimation for the Sterlitech HP4750 stirred cell is shown in the Supplementary Information, which yielded a value of 1.07 × 10^−5^ m/s for stirring at 120 RPM (see Figure S6). Figure 6 shows the change in the reverse salt flux coefficient (*B_f_*–*B*_0_) after contact with each alcohol for 5 min in each membrane. Figure 6 reveals that a large decrease is observed for the salt passage in the SW30XLE and XLE membranes, suggesting that the alcohol contact with these non-coated membranes might result in a densification of the active layer. This result agrees with the water permeance reduction observed with these membranes and suggests that active layers of non-coated membranes might be more susceptible to changes upon contact with short chain alcohols.

(15) $$B_{i}=J_{w,i}\frac{1-R_{i}}{R_{i}}\exp\left(-\frac{J_{w,i}}{k}\right)$$

**Figure 7:**
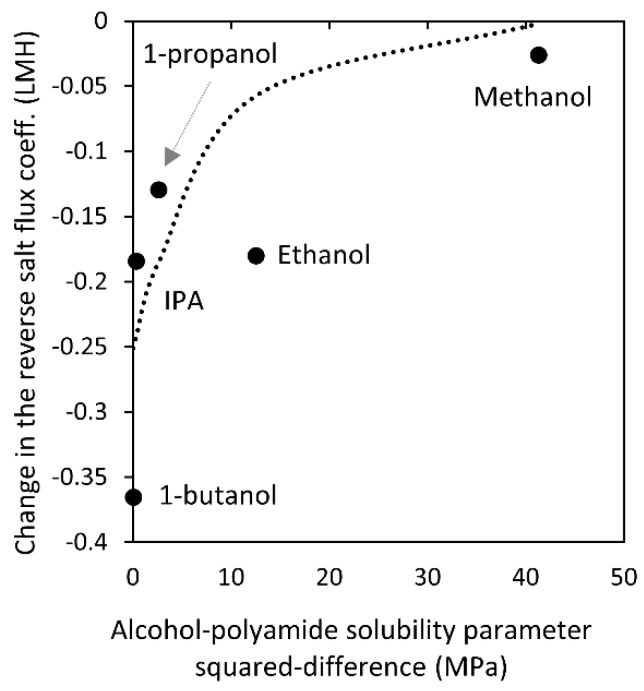
Calculated change in the reverse salt flux coefficient using Equation (15) versus the squared-difference in the reported solubility parameters of alcohols (Table 2) and fully-aromatic polyamide [18]. The dashed curve is a guide for the reader′s eye.

To identify a possible reason for the changes in water permeance of all membranes and reverse salt flux coefficient of the SW30XLE and XLE membranes, we investigated the relationship between these changes and different membrane and alcohol properties. Initially, we used the solubility parameter (*δ*) of the polyamide layer (PA) and the alcohols assuming that more favorable interactions (i.e., (*δ*_PA_- *δ*_alcohol_)^2^ → 0) would lead to larger changes in water permeance and reverse salt flux. The studies made by Aharoni indicate that aromatic polyamide layers have *δ*_PA_ *=* 23 MPa^1/2^ [18], suggesting that smaller property changes of the polyamide active layers should be seen after contact with methanol and larger after contact with 1-butanol, as the alcohol solubility parameter approaches the value of the polyamide layer (see Table 2). This trend correlates to the observed average change in reverse salt flux coefficient of the membranes as shown in Figure 7. However, it does not explain the large water permeance changes obtained after contact with methanol. Therefore, we concluded that polyamide-alcohol interactions result in a reduction in the reverse salt flux due to a densification of the polyamide layer. We propose this densification occurs when water (non-solvent) contacts the polyamide layer after the thin layer is softened due to alcohol contact, as shown by AFM nanoindentation experiments by Shin et al. [16]. Nevertheless, direct polyamide–alcohol interactions do not fully explain changes in the water permeance results after 5 min of contact with alcohol.

Following the study of polymer-alcohol interactions, we considered the PVOH-alcohol interactions, as PVOH is a commonly reported coating for TFC RO membranes, and is likely used for membranes SW30HRLE and SW30XLE based on ATR-FTIR and XPS data [29,31]. Hansen reported a wide range of solubility parameters for different protective PVOH films, calculated to be from 21 to 27 MPa^1/2^ from thinner to thicker coatings. Assuming a PVOH protective coating with a solubility parameter *δ*_PVOH_ = 27 MPa^1/2^ would still predict more favorable interactions between PVOH and ethanol compared to PVOH and methanol. However, methanol led to a higher increase water permeance compared to ethanol. Therefore, we believe that modification or removal of PVOH is not the main cause of the large changes in water permeance after methanol contact. Other alcohol characteristics that we considered but that did not provide high correlation to changes in transport properties were dipole moment, molecule dimensions and molecular weight, and surface tension (values listed in Table S2). 

**Figure 8:**
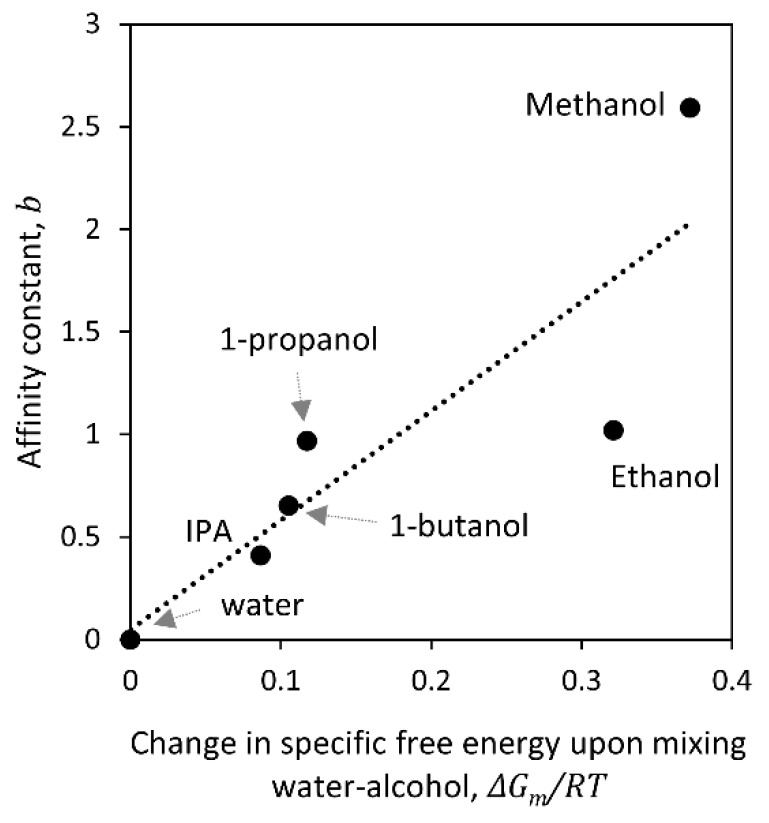
Affinity constant calculated from Equation (9) using the fitted *K_2_* for each alcohol versus the maximum change in Gibbs free energy of mixing (area under the curve in Equation (16)). The dotted line is a guide for the reader′s eye.

Using the binary interaction parameters between the alcohols tested in this work and water reported by Park et al. [42], and the UNIQUAC excess Gibbs free energy model [43], we calculated the change in the specific Gibbs free energy upon mixing water and alcohol using Equation (16) [44], as shown in Figure S7. Then, we estimated the maximum change and used it as a measure of the water-alcohol interactions. Figure 8 shows the affinity constant (*b*) calculated from Equation (9) using the fitted *K_2_* for each alcohol versus the maximum change in the specific Gibbs free energy of mixing water and alcohol. The latter was calculated as the area under the curve represented by Equation (16) and shown in Figure S7. Results show a positive linear correlation between these variables with a *R^2^* = 75.8%. Ethanol deviates most from the linear trend. We attribute this to the mathematical fitting because, in almost every case, the dual-mode sorption model underestimated the change in water permeance after ethanol contact, as shown in Figure 5. In general, alcohol and water interactions provided the highest correlation with the change in water permeance after contact among all properties studied. Our findings suggest that the energy released by mixing water and alcohol is translated into an increase in water permeance of TFC membranes. This result could be attributed to the breaking and exposing of hydrogen bond sites as suggested by Louie et al. [14]. However, this would be enabled by the energy released from alcohol interacting with water, rather than the alcohol interacting with the active layer leading to plasticization.

(16) $$\frac{\Delta G_{m}}{RT}=\frac{G^{E}}{RT}+\sum^{\text{​}}x_{i}\ln\gamma_{i}$$

**Figure 9:**
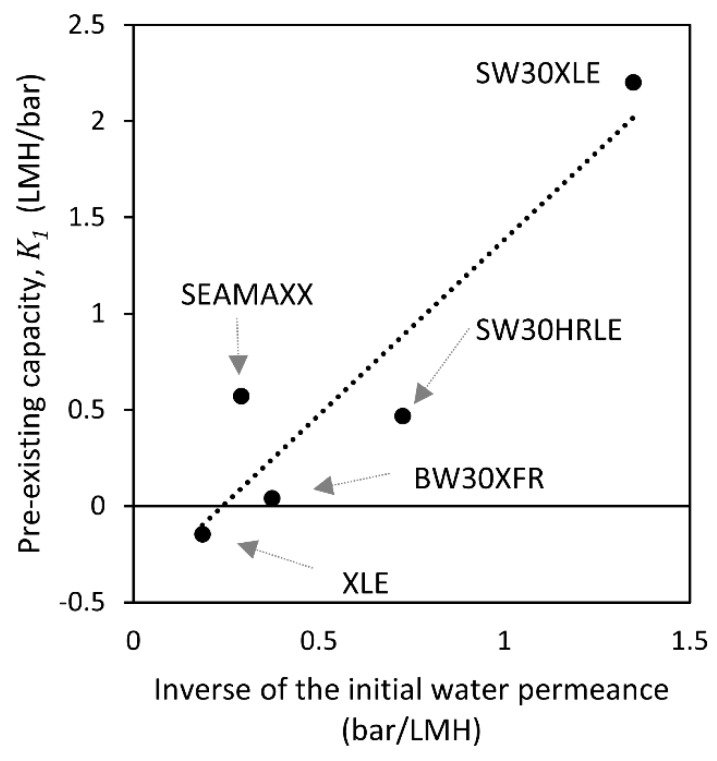
Estimated pre-existing capacity (at 5 min of contact) of the active layer from the dual-mode sorption model versus the inverse of the initial water permeance for each membrane. The dotted line is a guide for the reader′s eye.

Finally, we studied the active layer pre-existing capacity (*K*_1_), which is expected to be a characteristic of the polyamide layer itself at equilibrium. Figure 9 shows a linear correlation between the active layer pre-existing capacity (at 5 min of contact) and the inverse of the initial water permeance (*R^2^* = 86.1%). This result suggests that the change in water permeance after contact with the alcohol is higher if the membrane is tighter (such as a seawater desalination membrane) compared to a looser membrane with higher initial water permeance (such as a brackish water desalination membrane). If we assume that a membrane with higher cross-link density has more amide bonds and lower water permeance, then we can expect this membrane to be more susceptible to the formation of free volume by breaking hydrogen bonds between amide linkages, similarly to the example shown by Louie et al. [14]. Then, the negative pre-existing capacity for the XLE membrane suggests that XLE has a high initial free volume, and a short-term contact with alcohols leads to the formation of hydrogen bonds between amide linkages. 

**Figure 10:**
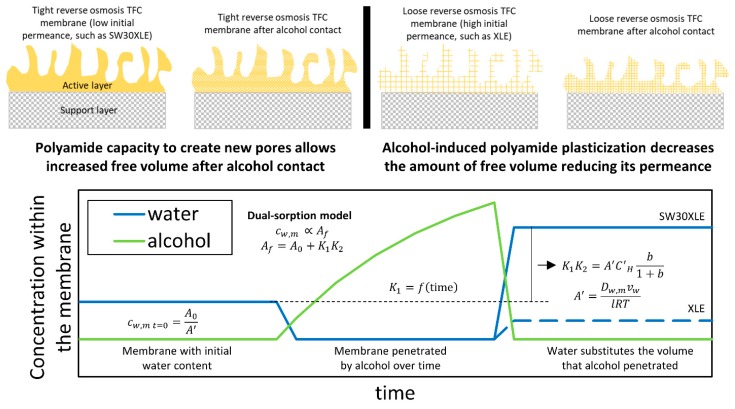
Schematic of the proposed change in tight and loose polyamide layers after contact with short chain alcohols. (**Top**) A comparison of the effect of alcohol contact between a tight and a loose TFC RO membranes. (**Bottom**) A representation of a three-stage mechanism that leads to changes in water permeance after alcohol contact.

Therefore, we suggest that membranes with low initial water permeance will show higher changes in permeance after contact with an alcohol because their higher density of inter-chain hydrogen bonds can be converted into volume for water to occupy. Figure 10 (top) shows a pictorial representation of this process. In the case of an active layer with greater affinity for alcohol, a plasticization effect will lead to a densification of the polyamide layer. This results in increased salt rejection and no increase (or reduction, depending on its initial capacity) in the water permeance. Figure 10 (bottom) shows a representation of a three-stage mechanism that leads to changes in water permeance after alcohol contact. First, a membrane with an initial concentration of water in the active layer contacts an excess of alcohol, which takes the place of the water within the active layer. Due to the more favorable interactions between the active layer and the alcohol, the concentration of alcohol within the membrane is higher than the water concentration. The concentration of alcohol within the membrane will be higher as time increases until it reaches equilibrium; however, in this work, the time was kept short and constant at 5 min. Finally, when an excess of water is contacted with the alcohol-wetted membranes, water takes the place of the alcohol within the active layer. However, the water concentration within the active layer is now different than the initial water concentration, and generally higher. The change in water content within the active layer depends on the alcohol miscibility with water and the polymer initial condition, as described by the dual-mode sorption model used above.

## 5. Conclusions

The effect of contact with different short-chain (C1–C4) alcohols on the transport properties of MPD-TMC-based polyamide active layers of commercial thin-film composite reverse osmosis membranes has been studied. Changes to water permeance and salt rejection depend on the type of membrane and alcohol used. A simplified dual-mode sorption model shows that changes in water permeance depend on two coupled factors: active layer pre-existing capacity and the affinity of the alcohol with the active layer. Active layers with higher pre-existing capacity to create new pores had lower water permeance before alcohol contact, such as seawater desalination membranes. Alcohols with higher affinity with the active layer had lower Gibbs free energy of mixing with water. Our findings suggest that water interactions with the alcohol (miscibility) determine the amount of alcohol within the polymer, which later can lead to previously-proposed disruption of inter-chain hydrogen bonds increasing the free volume water can access within the polymer. Our studies provide a modeling framework to estimate the changes in transport properties after short-term contact with short-chain alcohols that is especially useful when selecting conditions for wetting the support layer of TFC membranes for osmotically-driven membrane processes.

## Figures and Tables

**Table 1:** Measured transport properties of the TFC membranes before contact with alcohol. Error represents one standard deviation among at least 25 measurements.

TFC Membrane	Water Permeance (LMH/bar)	NaCl Rejection (%)
SW30HRLE	1.37 ± 0.09	94.8% ± 1.7%
SW30XLE	0.74 ± 0.11	90.9% ± 3.2%
SEAMAXX	3.44 ± 0.21	95.7% ± 1.1%
BW30XFR	2.67 ± 0.14	96.7% ± 1.4%
XLE	5.36 ± 0.35	87.6% ± 1.9%

**Table 2:** Reported in [34] solubility parameters for each short chain alcohol.

Alcohol	Solubility Parameter (MPa)^1/2^
Methanol	29.41
Ethanol	26.52
n-propanol	24.60
IPA	23.58
n-butanol	23.20
Water	47.90

## Supplementary materials

The following are available online at www.mdpi.com/xxx/s1, Figure S1: SEM images of the top surface of the studied TFC membranes at 2k magnification, Figure S2: SEM images of the top surface of the studied TFC membranes at 10k magnification, Figure S3: Relative change in root-mean-square surface roughness after alcohol contact treatment. The dashed line shows the average of all membranes, and the error bars are calculated by propagating the error in each individual membrane measurement, Figure S4: Surface Young’s moduli after contact with water and the C1–C4 alcohols studied for each membrane tested. Error bars indicate standard deviation among 256 points on a surface area of 10 µm × 10 µm, Figure S5: Captive air bubble contact angle in water and the C1–C4 alcohols studied of the polyamide layers of the membranes, Figure S6: (Left) Schematic of the Sterlitech HP4750 stirred cell. Shown are the radius of the membrane active circular area (r2), the radius of the stir bar (r1), and the distance between the stir bar and the membrane surface (h). (Center) Normalized linear velocity profile at different radii (x-axis) and height (curves) positions. (Right) Mass-transfer coefficient and Reynolds number calculation assuming a rectangular flow channel of height h and width r2, Figure S7: Calculated change in Gibbs free energy upon mixing water and C1–C4 alcohols calculated using UNIQUAC and interaction parameters reported by Park et al. [42], Table S1: Literature review on alcohol contact and its effect on membrane productivity and selectivity, Table S2: Alcohol properties: dipole moment [45], molecular weight, molar surface area [46], molecular diameter [47], and surface tension [48], Table S3: Atomic content on the surface of the TFC membranes studied via XPS, Table S4: Root-mean-square roughness of the active layer of the rinsed membranes after contact with DI water for 5 min. Uncertainty represents one standard deviation over a least three different spots of an area of 5 μm × 5 μm, Table S5: Statistical analysis of the change in transport properties of TFC membranes before and after contact with DI water for 5 min and 2 h. Confidence interval is 95%, Table S6: Statistical analysis of the change in transport properties of TFC membranes before and after contact with ethanol for 5 min and 2 h. Confidence interval is 95%, Table S7: Statistical analysis of the change in transport properties of TFC membranes before and after contact with short chain alcohols for 5 min. Confidence interval is 95%, Table S8: Statistical analysis of the dual-sorption model fitting of the experimental data. Values in bold indicate model fittings that are statistically different than the experimental results at a confidence interval is 90%. Values in parentheses indicate the determination coefficient (*R*^2^) between the experimental and the model results at the same treatment (membrane type or alcohol); Appendix. Estimation of mass-transfer coefficient for Sterlitech HP4750 cell.
