# Supplementary material for: Effect of Short-Term Contact with C1–C4 Monohydric Alcohols on the Water Permeance of MPD-TMC Thin-Film Composite Reverse Osmosis Membranes
Source: Membranes (Basel). 2019 Jul 26;9(8):92. doi: 10.3390/membranes9080092 (PMC6723597; doi:10.3390/membranes9080092)
Supplement: Supplementary file 1 [file membranes-09-00092-s001.pdf]

Supplementary Materials

Figures

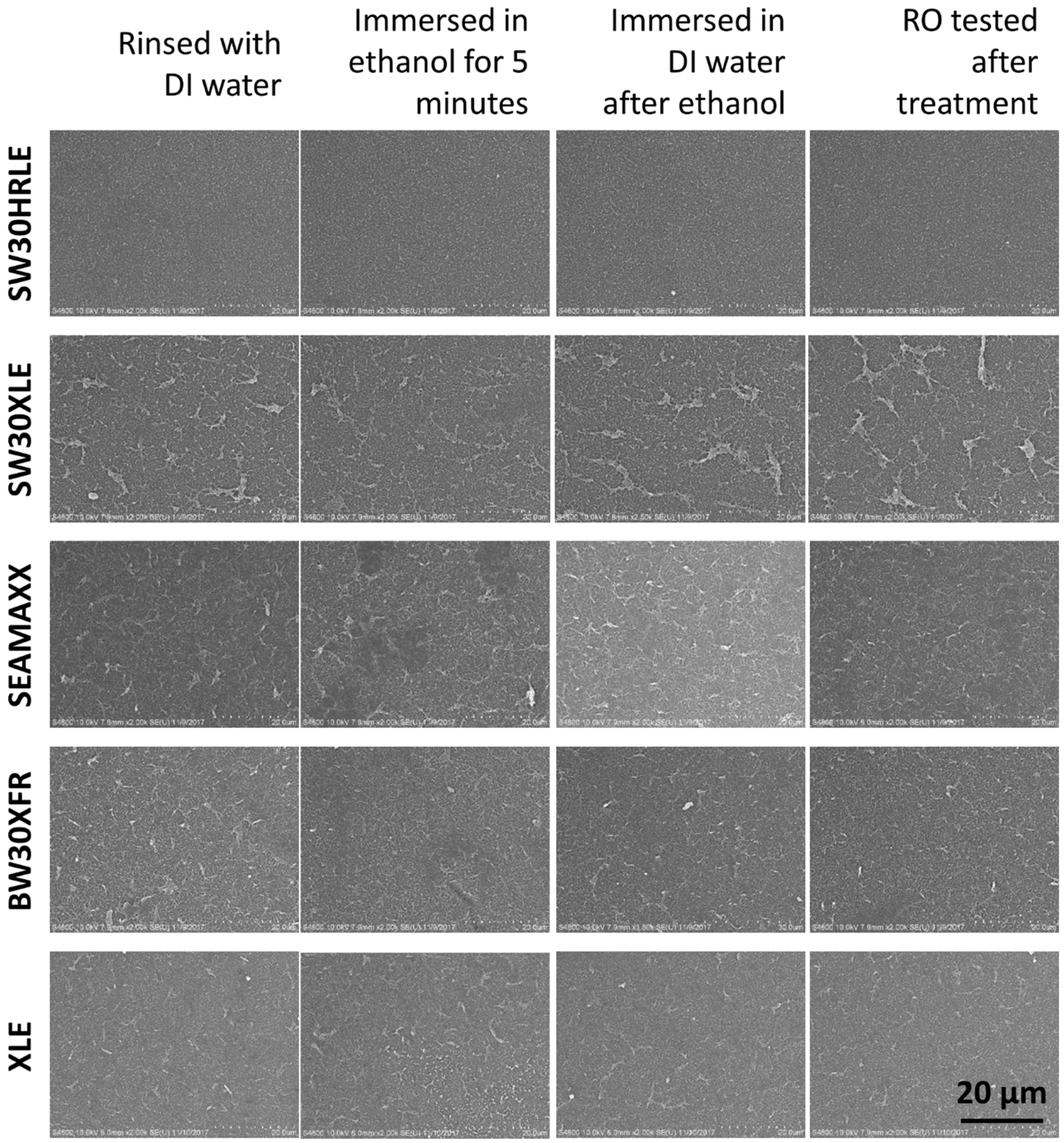

Figure S1. SEM images of the top surface of the studied TFC membranes at 2k magnification.

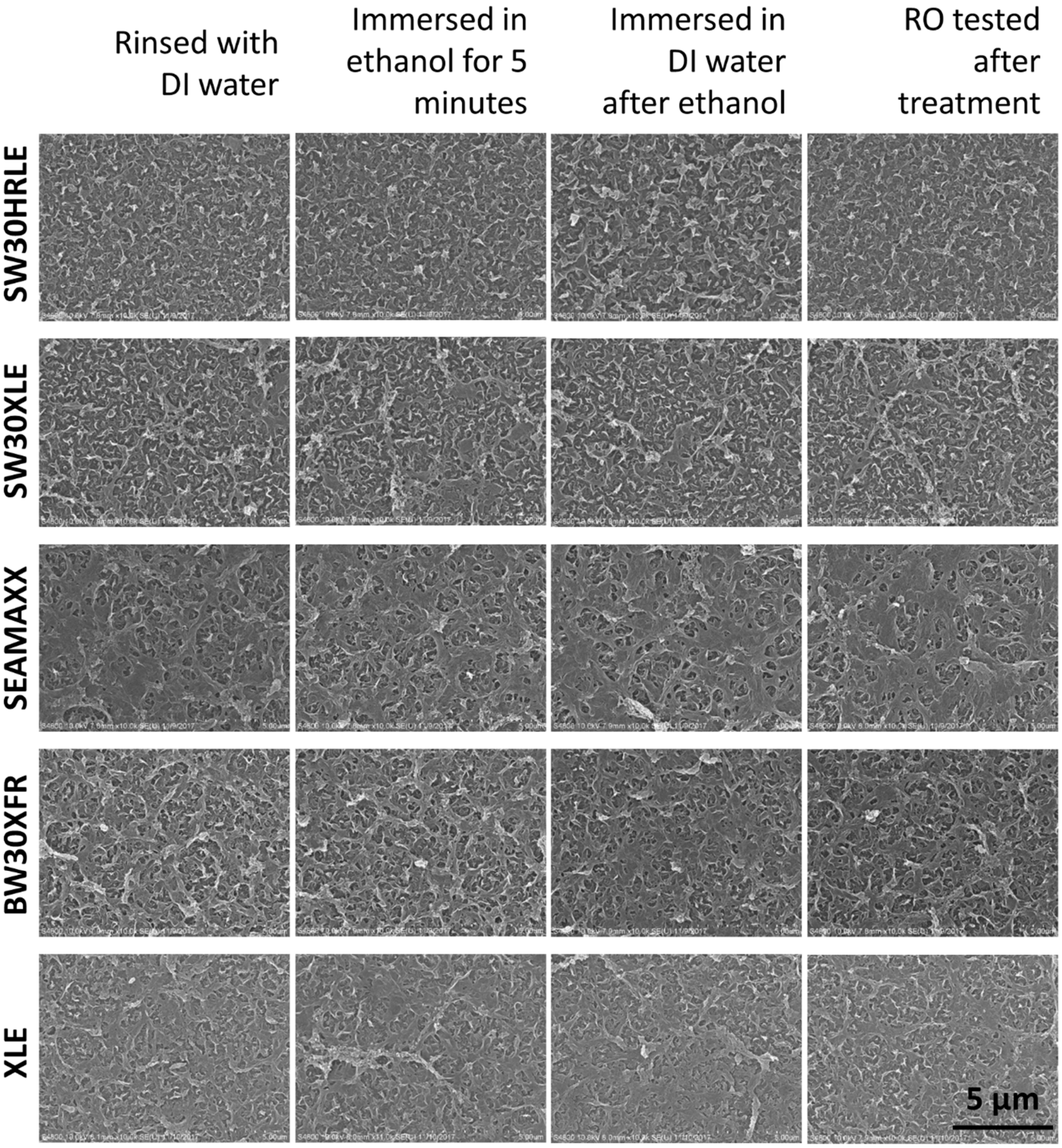

Figure S2. SEM images of the top surface of the studied TFC membranes at 10k magnification.

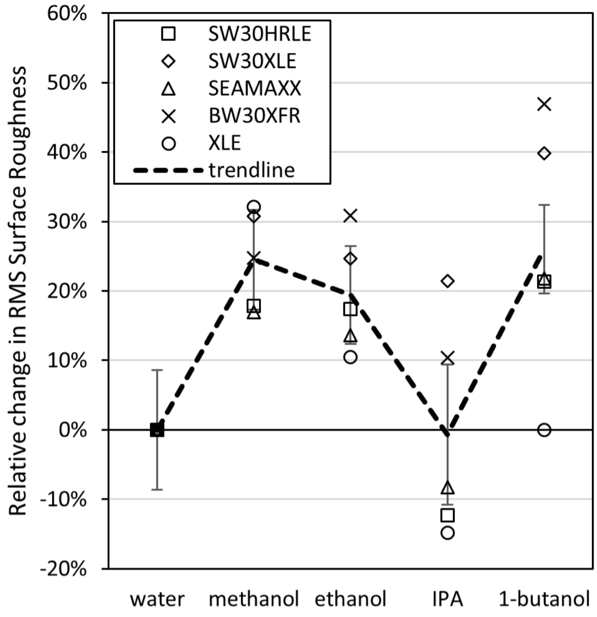

Figure S3. Relative change in root-mean-squared surface roughness after alcohol contact treatment. The dashed line shows the average of all membranes, and the error bars are calculated by propagating the error in each individual membrane measurement.

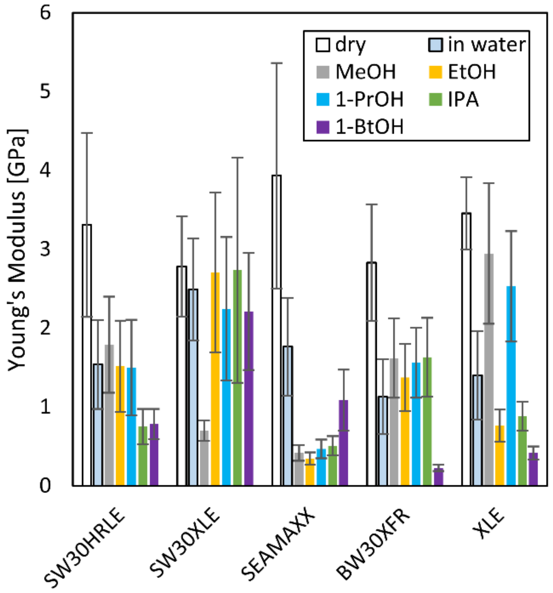

Figure S4. Surface Young's moduli after contact with water and the C1-C4 alcohols studied for each membrane tested. Error bars indicate standard deviation among 256 points on a surface area of 10 µm × 10 µm.

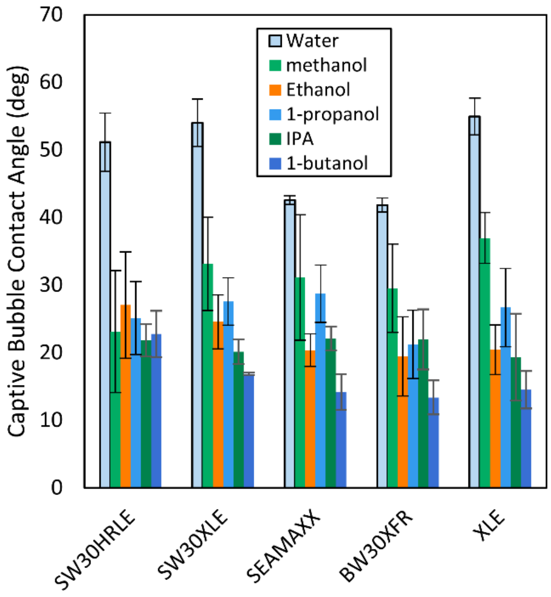

Figure S5. Captive air bubble contact angle in water and the C1-C4 alcohols studied of the polyamide layers of the membranes.

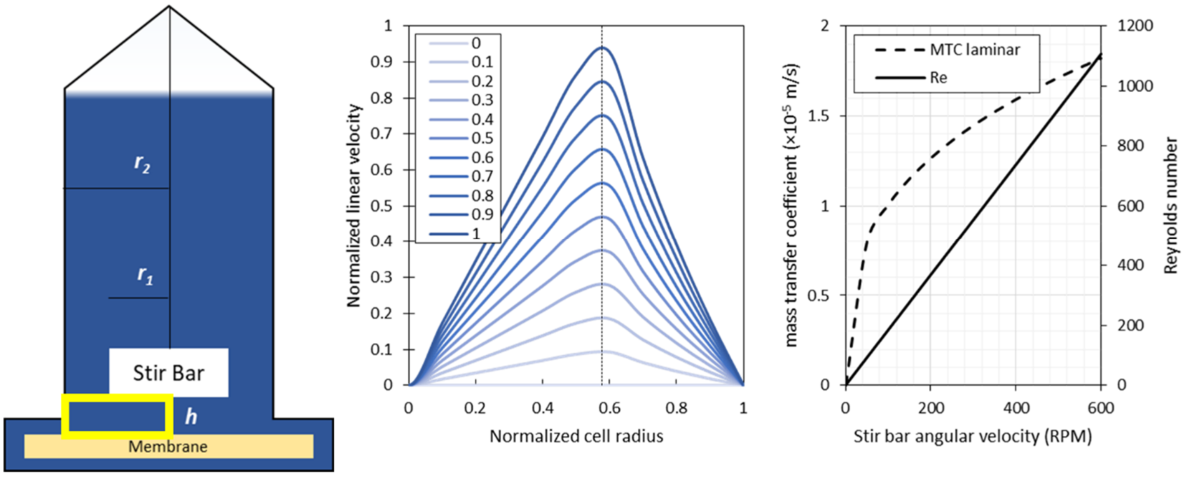

Figure S6. (Left) Schematic of the Sterlitech HP4750 stirred cell. Shown are the radius of the membrane active circular area (r_2), the radius of the stir bar (r_1), and the distance between the stir bar and the membrane surface (h). (Center) Normalized linear velocity profile at different radii

(x-axis) and height (curves) positions. (Right) Mass-transfer coefficient and Reynolds number calculation assuming a rectangular flow channel of height *h* and width r_2.

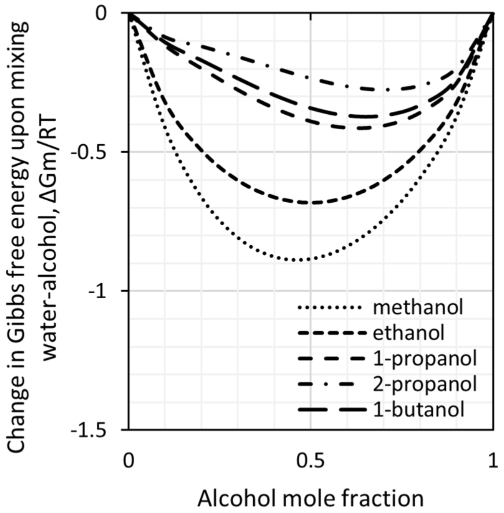

Figure S7. Calculated change in Gibbs free energy upon mixing water and C1-C4 alcohols calculated using UNIQUAC and interaction parameters reported by Park et al. Reference may be found in the main text.

Tables

Table S1. Literature review on alcohol contact and its effect on membrane productivity and selectivity.

Membrane	Alcohol	Method	Testing method	Effect on productivity	Effect on selectivity	Explanation of result	Reference in main text
HR95PP and HR98PP commercial MPD-TMC-based TFC RO	Aqueous solutions of ethanol and IPA	Membranes wetted with alcohols for defined times and later washing with DI water	Reverse osmosis filtration of 0.5 wt.% NaCl	Increased water flux	Uncompromised NaCl rejection	Similarity in solubility parameters of ethanol (26.6 MPa1/2) and IPA (23.6 MPa1/2) to the fully aromatic polyamide (23.0 MPa1/2) allowed interactions between the polyamide and the alcohol	9
Hand-cast TFC RO membranes with crosslinked poly(vinyl alcohol) (PVOH) as the active layer	20 wt% aqueous solutions of C1-C4	Membranes were immersed in alcohols for 2 h	Reverse osmosis filtration of 2000 ppm NaCl	Water flux of membranes increased by 50%	Unchanged NaCl rejection compared to untreated membranes	Observation in line with reported literature	15
Lab-made carbon molecular sieves of carbonized polyimide (Matrimid®, d = 29.96 MPa1/2 and P84, d = 36.80 MPa1/2) films	Linear monohydric alcohols (C1-C4)	Polyimide films immersed in alcohol for 24 h, followed by 24 h of drying, then carbonization	Gas permeation of pure N2, CH4, CO2 and O2	Decreased gas flux	Improved CO2/CH4 selectivity	Changes in the polyimide films (and the properties of the molecular sieves made out of these) are dependent on size of the alcohol, composition of the polymer, free volume in the polymer (especially before treatment), and interactions between polymer and alcohol	12
Commercial SWC4 TFC RO membrane	Ethanol (for studies on both water flux and salt rejection)	Membranes immersed in alcohol for 5 min	Reverse osmosis filtration of 1600 ppm NaCl	Increased water permeance	Increased NaCl rejection when drying was not allowed	swelling of the active layer occurs, which may disrupt inter-chain interactions, such as hydrogen bonding, creating space for water to penetrate into the membrane	14
Commercial TFC RO membranes (SW30XLE and BW30)	IPA	Immersion of membranes into IPA for 1 h, followed by polydopamine coating	Reverse osmosis filtration of 2000 ppm NaCl	Increased water flux for the coated SW30XLE membrane but decreased for the coated BW30 membrane	Increased NaCl rejection for the coated SW30XLE membrane but decreased for the coated BW30 membrane	Denser porous support structure of BW30 compared to SW30XLE led to opposite behavior in water flux and NaCl rejection after coating	7

Table S1. Literature review on alcohol contact and its effect on membrane productivity and selectivity (*continued*).

**Membrane**	**Alcohol**	**Method**	**Testing method**	**Effect on productivity**	**Effect on selectivity**	**Explanation of result**	**Reference in main text**
Lab-made hollow fiber TFC membranes. Torlon® substrate. Four amines (including MPD) were reacted with TMC to create active layers	Methanol	Immersion of membranes into methanol for 2 min	Pervaporation of a 85 wt% IPA solution circulated through the shell side	Increased water passage during pervaporation (IPA dehydration)	Decreased separation factor between water and IPA	Reduced thickness, i.e. distance for diffusion, led to higher water permeability	13
Commercial TFC RO membranes (including BW30 and SW30HR)	Ethanol	Presoaked the membranes in DI water, immersed them into ethanol for 24 h, re-immersed them in DI water	Forward osmosis with draw solution 1.5M NaCl (or 1.5M MgSO4), and feed of DI water	increased osmotic water flux and for all the membranes wetted with alcohol compared to control experiments	increased reverse salt flux for all the membranes wetted with alcohol compared to control experiments	Results are a combined effect of a removal of the coating layer and improved wetting of the polysulfone support	10
Hand-cast MPD-TMC TFC membranes with PDA coating, and polyacrylonitrile support	Methanol and ethanol	Immersion for 2 days	Pressure-retarded osmosis with draw solution 3.5 wt% NaCl, DI water as feed, and 0 bar of hydraulic pressure	increased water permeance, with methanol yielding higher increases from 1 to 24 h	increased reverse salt flux	Removal of material from the active layer and increased free volume of the polyamide layer after ethanol treatment. Methanol high polarity and smaller molecule size yielded higher increases	11
Hand-cast TFC membranes with an MPD-TMC-based	Ethanol and 50% IPA	Immersed membranes in water, then in alcohol for 5 min, and washed and immersed membranes into water	Forward osmosis with draw solution 1M NaCl and feed of DI water	Osmotic water flux increased after treatment, with ethanol yielding higher increases than aqueous IPA	Salt flux increased after treatment, with ethanol yielding higher increases than aqueous IPA	The capacity of ethanol and IPA to swell fully-aromatic polyamide layers	8
Hand-cast MPD-TMC active layers on plasma-treated porous polyethylene supports	Ethanol, IPA and Benzyl Alcohol	Alcohol poured on the membrane surface, then contact from 1 to 10 min. Then, rinsing with DI water	Reverse osmosis filtration of 2000 ppm NaCl	Alchohols led to increased water permeance, with BA yieding the highest increase	Unchanged NaCl rejection	Optimal solvent-polyamide interactions (defined by difference in Hansen parameters, Ra) between BA and polyamide (8.1 MPa), compared to the high Ra values with ethanol (12.7 MPa) and IPA (11.2 MPa)	16

Table S2. Alcohol properties: dipole moment, molecular weight, molar surface area, molecular diameter, and surface tension. References may be found in the main text.

Alcohol	Dipole moment (Debye)	Molecular weight (g/mol)	Molar surface area (×10^8 cm^2/mol)	Molecular diameter (nm)	Surface Tension (erg/cm^2)
Methanol	1.70	32.04	3.987	0.41	24.8 (264 K)
Ethanol	1.69	62.07	8.052	0.52	24.0 (270 K)
n-propanol	1.68	92.09	17.41		25.4 (273 K)
IPA	1.66	92.09	20.68	0.58	23.1 (270 K)
n-butanol	1.66	122.12			26.1 (265 K)
Water	1.85	18.01	0.7225	0.26	77.0 (270 K)

Table S3. Atomic content on the surface of the TFC membranes studied via XPS.

Membrane	at. % Carbon	at. % Oxygen	at. % Nitrogen
SW30HRLE	71.4% ± 0.7%	19.9% ± 2.9%	8.5% ± 2.1%
SW30XLE	70.8% ± 0.8%	22.2% ± 0.9%	6.9% ± 0.2%
SEAMAXX	73.2% ± 0.6%	14.3% ± 0.4%	12.4% ± 0.8%
BW30XFR	73.7% ± 0.5%	13.3% ± 0.3%	12.9% ± 0.4%
XLE	74.7% ± 0.3%	13.3% ± 0.3%	11.9% ± 0.2%

Table S4. Root-mean-squared roughness of the active layer of the rinsed membranes after contact with DI water for 5 minutes. Uncertainty represents one standard deviation over a least 3 different spots of an area of 5 μm × 5 μm.

	SW30HRLE	SW30XLE	SEAMAXX	BW30XFR	XLE
RMS Roughness (nm)	49.6 ± 8.8	45.7 ± 11.2	43.1 ± 5.7	41.8 ± 8.8	36.2 ± 6.4

Table S5. Statistical analysis of the change in transport properties of TFC membranes before and after contact with DI water for 5 min and 2 h. Confidence interval is 95%.

TFC Membrane	Time	Permeance	Rejection
SW30HRLE	5 min	No significant change	No significant change
SW30HRLE	2 h	No significant change	No significant change
SW30XLE	5 min	No significant change	No significant change
SW30XLE	2 h	No significant change	No significant change
SEAMAXX	5 min	No significant change	Increases
SEAMAXX	2 h	Decreases	No significant change
BW30XFR	5 min	Decreases	Increases
BW30XFR	2 h	No significant change	Increases
XLE	5 min	Decreases	Increases
XLE	2 h	Decreases	Increases

Table S6. Statistical analysis of the change in transport properties of TFC membranes before and after contact with ethanol for 5 min and 2 h. Confidence interval is 95%.

TFC Membrane	Wetting	Permeance	Rejection
SW30HRLE	5 min	Increases	Increases
SW30HRLE	2 h	Increases	Increases
SW30XLE	5 min	Increases	Increases
SW30XLE	2 h	Increases	Increases
SEAMAXX	5 min	Increases	No significant change
SEAMAXX	2 h	Increases	Decreases
BW30XFR	5 min	Increases	Increases
BW30XFR	2 h	Increases	No significant change
XLE	5 min	Increases	Increases
XLE	2 h	No significant change	No significant change

Table S7. Statistical analysis of the change in transport properties of TFC membranes before and after contact with short chain alcohols for 5 min. Confidence interval is 95%.

TFC Membrane	Wetting	Permeance	Rejection
SW30HRLE	Methanol	Increases	Increases
SW30HRLE	Ethanol	Increases	Increases
SW30HRLE	1-Propanol	Increases	Increases
SW30HRLE	Isopropanol	Increases	Increases
SW30HRLE	1-Butanol	Increases	Increases
SW30XLE	Methanol	Increases	Increases
SW30XLE	Ethanol	Increases	Increases
SW30XLE	1-Propanol	Increases	Increases
SW30XLE	Isopropanol	Increases	Increases
SW30XLE	1-Butanol	Increases	Increases
SEAMAXX	Methanol	Increases	Decreases
SEAMAXX	Ethanol	Increases	No significant change
SEAMAXX	1-Propanol	Increases	No significant change
SEAMAXX	Isopropanol	Increases	Increases
SEAMAXX	1-butanol	No significant change	Increases
BW30XFR	Methanol	No significant change	Decreases
BW30XFR	Ethanol	Increases	Increases
BW30XFR	1-Propanol	No significant change	No significant change
BW30XFR	Isopropanol	No significant change	Increases
BW30XFR	1-Butanol	Decreases	No significant change
XLE	Methanol	No significant change	Increases
XLE	Ethanol	Increases	Increases
XLE	1-Propanol	No significant change	Increases
XLE	Isopropanol	No significant change	Increases
XLE	1-Butanol	Decreases	Increases

Table S8. Statistical analysis of the dual-sorption model fitting of the experimental data. Values in bold indicate model fittings that are statistically different than the experimental results at a confidence interval is 90%. Values in parenthesis indicate the determination coefficient (R^2) between the experimental and the model results at the same treatment (membrane type or alcohol).

**t-statistic**	MeOH (99%)	EtOH (99%)	1-PrOH (99%)	IPA (99%)	1-BtOH (96%)
SW30HRLE (93%)	0.98	0.50	0.99	0.90	0.06
SW30XLE (99%)	0.37	0.89	0.29	0.16	0.07
SEAMAXX (84%)	0.73	0.76	0.54	0.42	0.90
BW30XFR (25%)	1.00	**1.70**	0.80	0.77	**2.22**
XLE (13%)	0.37	0.93	0.15	0.07	1.68

Estimation of mass transfer coefficient for Sterlitech HP4750 cell

To estimate the mass-transfer coefficient, we assumed a rectangular channel of height h and width r_2 as shown in Figure S2 (Left). We also assumed the stir bar to be a cylinder. We defined an average linear velocity through the channel in cylindrical coordinates using Eq. S1. A non-slip boundary condition was applied to the surface of the membrane, the walls of the cell, and the stir bar. With these conditions, we solved the equation of motion to calculate the linear velocity at each position within the rectangular channel.

$$\hat{v} = \frac{\int_0^{r_2} r \int_0^h v dz dr}{\int_0^{r_2} r \int_0^h dz dr} \quad \text{(S1)}$$

$$v = \begin{cases} \frac{\omega z}{h} r & 0 < r \leq r_1 \\ \frac{\omega z}{h}\left(\frac{r_1^2}{r_2^2 - r_1^2}\right)\left(\frac{r_2^2}{r} - r\right) & r_1 < r \leq r_2 \end{cases} \quad \text{(S2)}$$

Eq. S2 shows the linear velocity formula within the rectangular channel, where r_1 is the radius of the stir bar and ω is the angular velocity of the stir bar. Figure S2 (Center) shows the normalized linear velocity profile at different radii (x-axis) and height (curves) positions. Normalized values

were calculated by dividing the linear velocity by the maximum velocity ($v_{max}= \omega r_1$), the radius by the radius of the membrane active circular area, and the height position by the distance between the stir bar and the membrane surface.

$$\hat{v} = \frac{\omega}{{r_2}^2}\left(\frac{{r_1}^3}{3} + \left(\frac{{r_1}^2}{{r_2}^2-{r_1}^2}\right)\left({r_2}^2(r_2 - r_1) - \frac{{r_2}^3-{r_1}^3}{3}\right)\right) \tag{S3}$$

Eq. S2 was substituted into Eq. S1 to obtain Eq. S3 which is the linear average velocity in the rectangular channel. It depends on the angular velocity (set by a stirring plate) and the stir bar and active area radii. This average linear velocity was used to calculate the Reynolds number as shown in Eq. S4. The hydraulic diameter (d_H) was calculated using Eq. S5. The Schmidt number (Sc) and the Sherwood number (Sh, for laminar flow in a rectangular channel [S1]) are shown in Eq. S6 and S7. The length of the channel (L) was analytically determined to be $1.33\pi r_2$. The mass-transfer coefficient (k) is calculated using the definition of the Sherwood number, and shown in Eq. S8. Figure S2 (Right) shows the calculated mass-transfer coefficient and Reynolds number at different stir bar angular velocities in the Sterlitech HP4750 stirred cell. The properties used were: viscosity of the solution (μ) 8.9×10^{-4} Pa, density of the solution (ρ) 1000 kg/m^3, and diffusivity of sodium chloride in water (D) 1.6×10^{-9} m^2/s [S2].

$$\mathrm{Re} = \frac{d_H \hat{v} \rho}{\mu} \tag{S4}$$

$$d_H = \frac{2hr_2}{h+r_2} \tag{S5}$$

$$\mathrm{Sc} = \frac{\mu}{\rho D} \tag{S6}$$

$$\mathrm{Sh} = 1.85\left(\mathrm{ReSc}\frac{d_H}{L}\right)^{0.33} \tag{S7}$$

$$k = \frac{\mathrm{Sh}D}{d_H} \tag{S8}$$

[S1] J.R. McCutcheon, M. Elimelech, Influence of concentrative and dilutive internal concentration polarization on flux behavior in forward osmosis, J. Memb. Sci. 284 (2006) 237–247.

[S2] G.M. Geise, H.B. Park, A.C. Sagle, B.D. Freeman, J.E. McGrath, Water permeability and water/salt selectivity tradeoff in polymers for desalination, J. Memb. Sci. 369 (2011) 130–138.
